# Satellite Jitter Estimation and Validation Using Parallax Images

**DOI:** 10.3390/s17010083

**Published:** 2017-01-02

**Authors:** Jun Pan, Chengbang Che, Ying Zhu, Mi Wang

**Affiliations:** 1 The State Key Laboratory of Information Engineering in Surveying, Mapping and Remote Sensing, Wuhan University, 129 Luoyu Road, Wuhan 430079, China; panjun1215@whu.edu.cn (J.P.); ccb@whu.edu.cn (C.C.); yzhu1003@whu.edu.cn (Y.Z.); 2 Collaborative Innovation Center for Geospatial Technology, 129 Luoyu Road, Wuhan 430079, China

**Keywords:** satellite jitter, parallax images, jitter displacement, jitter estimation, jitter validation

## Abstract

Satellite jitter (SJ) is an important error source that affects the geometric accuracy of high resolution satellite imagery. In this paper, the quantitative relationship between the jitter displacement (image displacement caused by SJ) and relative registration error obtained from parallax images is deduced to be theoretical in detail, and the jitter displacement estimation model is built to estimate the jitter displacement. Then, a simulation experiment is carried out to validate the feasibility of using the built jitter displacement estimation model to estimate the jitter displacement. Finally, experiments with real images in DengFeng (China) including multispectral images of Ziyuan-3 (ZY-3) satellite and high resolution (HR) images of Ziyuan1-02C (ZY1-02C) satellite are used to validate the effectiveness of utilizing the built jitter displacement estimation model to do the jitter estimation. High accuracy ground reference data are further used to evaluate the accuracy of the estimation. Experimental results show that the average estimated error for jitter displacement of ZY-3 is 2.96% and 0.11% in amplitude and frequency respectively, and the estimated error for jitter displacement of ZY1-02C is 8.46% and 0.35% in amplitude and frequency, respectively.

## 1. Introduction

With increased spatial resolution of satellite images, the influence of satellite jitter (SJ) on the imaging quality becomes more and more obvious, and seriously limits the application of satellite image products [1,2,3,4,5]. For instance, Ayoub et al. found that the unmodelled jitter of QucikBird produced an approximately 5 pixels (2.5 m) geometric distortion mainly around 1 Hz and an approximately 0.2 pixels (0.1 m) geometric distortion mainly around 4.3 Hz [6]. Takaku and Tadono encountered two kinds of systematic waving noise in some Digital Surface Model generated from ALOS PRISM processing and examined their relations to attitude fluctuations [7]. Moreover, SJ has a great influence on internal and external calibration of cameras during geometric preprocessing [8,9]. Therefore, the estimation of SJ is more essential and significant because it is the foundation of the compensation for image distortions caused by SJ.

Much research on utilizing parallax images to estimate SJ has been performed under circumstances lacking high accuracy satellite attitude measuring instruments (star-tracker and gyro). Roques et al. proposed a method to identify micro-vibrations of satellite platforms in pitch, row and yaw—three directions—using stereo images [10]. Teshima and Iwasaki estimated attitude fluctuation between two different bands of ASTER short-wave infrared images and found a SJ with a frequency of about 1.5 Hz [11]. Mattson et al. reconstructed a jitter series for High Resolution Imaging Science Experiment (HiRISE) onboard the Mars Reconnaissance Orbiter (MRO) using staggered Charge-Coupled Devices (CCDs) [12]. During the in-flight commissioning period of PLEIDES-HR satellites, Amberg et al. estimated the attitude perturbances caused by SJ by exploiting multispectral CCD arrays [13]. Jiang et al. considered the impact of SJ on Ziyuan1-02C (ZY1-02C) imaging and used staggered CCDs to restore the high-frequency attitude based on a rigorous geometric model [14]. Sun et al. used eight staggered CCD arrays to detect the satellite jitter of Chinese mapping satellite-1 [15]. Tong et al. and Zhu et al. also presented several jitter detection methods based on multispectral images, stereo images and panchromatic images, and validated them by ZY-3 images [5,16,17,18,19].

In this work, a jitter displacement (image displacement caused by SJ) estimation model was built according to the deduced quantitative relationship between the jitter displacement and relative registration error obtained from parallax images. Both simulation experiments and real image experiments were carried out to validate the feasibility and effectiveness of utilizing the built jitter displacement estimation model to do the jitter estimation.

## 2. Methodology

### 2.1. Jitter Displacement Estimation Modelling

The principle of jitter displacement estimation is based on the design of satellite CCD sensors with parallax observation systems. In the parallax observation system, each linear sensor scans the same ground feature at different times and an object will be imaged at the same location in different images but at different times. However, some exterior orientation elements of the camera used in the observation system might have some small changes due to SJ during the observing interval time, and thus the same object might be imaged at slightly different locations in different images [17,20]. Thus, SJ can be reflected by relative registration error obtained from parallax images and the jitter displacement might be estimated by the relative registration error.

According to theory of Fourier transform, SJ can be converted into the attitude jitter component combined by several sinusoidal functions with different amplitudes, frequencies and phases [21], as shown in the following:

(1) $$\varphi (t)=\sum_{i=1}^{\infty }A_{i}\sin(2\pi f_{i}t+\varphi_{i})$$

where φ(t) is the jitter component of satellite attitude at imaging time t, and $A_{i}$, $f_{i}$, $\varphi_{i}$ are the amplitude, frequency and phase of the *i*th component, respectively. In order to simplify the derivation, it is assumed that the satellite platform contains only a single frequency jitter as follows:

(2) φ(t)=Asin(2πft+φ)

Considering the angle of attitude fluctuation caused by SJ is small, the jitter displacement, d(t), can be calculated based on photogrammetric theory according to Equation (3):

(3) $$\begin{array}{cc} d(t) & =f_{c}\cdot \varphi (t)/p\\ & =A_{d}\cdot \sin(2\pi ft+\varphi_{d}) \end{array}$$

where d(t) is jitter displacement, $f_{c}$ is the focal length of the camera, p is the pixel size in focal plane; $A_{d}$, f, $\varphi_{d}$ are the amplitude, frequency and phase of jitter displacement and $A_{d}=f_{c}\cdot A/p$, $\varphi_{d}=\varphi$.

According to the above-mentioned principle of jitter estimation using parallax images, relative registration error between parallax images with some imaging time interval can be denoted as

(4) r(t)=d(t+Δt)−d(t)

where r(t) is the relative registration error. By substituting Equation (3) into Equation (4), there is

(5) $$r(t)=A_{d}\cdot (\sin(2\pi f(t+\Delta t)+\varphi_{d})-\sin(2\pi ft+\varphi_{d}))$$

SJ is time varying, so the relative registration error caused by SJ is also varying with the imaging time interval of parallax images. When Δt=nT (n∈N, T is the minimum positive period of SJ and T=1/f), the jitter displacement of two parallax images are same, which means that the relative registration error will be zero and the SJ cannot be estimated by it. When Δt≠nT, it is enough to just analyze r(t) in the minimum positive period, i.e., 0<fΔt=Δt/T<1. Thus, Equation (5) can be expanded as

(6) $$\begin{array}{cc} r(t) & =A_{d}\cdot (\sin(2\pi ft+\varphi_{d}+2\pi f\Delta t)-\sin(2\pi ft+\varphi_{d}))\\ & =A_{d}\cdot (\sin(2\pi ft+\varphi_{d})\cos(2\pi f\Delta t)+\cos(2\pi ft+\varphi_{d})\sin(2\pi f\Delta t)-\sin(2\pi ft+\varphi_{d})) \end{array}$$

Let sin(2πfΔt)=a, cos(2πfΔt)=b, so $a^{2}+b^{2}=1$. According to the theory of trigonometric function, Equation (6) can be simplified as

(7) $$\begin{array}{cc} r(t) & =A_{d}\cdot \left(\left(b-1)\sin(2\pi ft+\varphi_{d})+a\cos(2\pi ft+\varphi_{d}\right)\right)\\ & =A_{d}\cdot \sqrt{{\left(b-1\right)}^{2}+a^{2}}(\frac{b-1}{\sqrt{{\left(b-1\right)}^{2}+a^{2}}}\sin(2\pi ft+\varphi_{d})+\frac{a}{\sqrt{{\left(b-1\right)}^{2}+a^{2}}}\cos(2\pi ft+\varphi_{d}))\\ & =A_{d}\cdot \sqrt{2-2b}\sin(2\pi ft+\varphi_{d}+\theta ) \end{array}$$

where $\sin\theta =\frac{a}{\sqrt{{\left(b-1\right)}^{2}+a^{2}}},\cos\theta =\frac{b-1}{\sqrt{{\left(b-1\right)}^{2}+a^{2}}}$. Substituting cos(2πfΔt)=b into Equation (7), there is

(8) $$r(t)=A_{d}\cdot \sqrt{2-2\cos(2\pi f\Delta t)}\sin(2\pi ft+\varphi_{d}+\theta )$$

Let $A_{r}=A_{d}\cdot \sqrt{2-2\cos(2\pi f\Delta t)}$, $\varphi_{r}=\varphi_{d}+\theta$, there is

(9) $$r(t)=A_{r}\cdot \sin(2\pi ft+\varphi_{r})$$

where $A_{r}$, $\varphi_{r}$ is the amplitude and phase of relative registration error. According to Equations (8) and (9), the quantitative relationship between $A_{d}$ and $A_{r}$ is given as

(10) $$A_{d}=A_{r}/\sqrt{2-2\cos(2\pi f\Delta t)}=A_{r}/(2\sin(\pi f\Delta t))$$

As to θ, there is

(11) $$\begin{array}{cc} \sin\theta & =\frac{\sin(2\pi f\Delta t)}{\sqrt{{\left(\cos(2\pi f\Delta t)-1\right)}^{2}+\sin^{2}(2\pi f\Delta t)}}=\frac{2\sin(\pi f\Delta t)\cdot \cos(\pi f\Delta t)}{\sqrt{4\sin^{2}(\pi f\Delta t)}}\\ & =\cos(\pi f\Delta t) \end{array}$$

(12) $$\begin{array}{cc} \cos\theta & =\frac{\cos(2\pi f\Delta t)-1}{\sqrt{{\left(\cos(2\pi f\Delta t)-1\right)}^{2}+\sin^{2}(2\pi f\Delta t)}}=\frac{-2\sin^{2}(\pi f\Delta t))}{\sqrt{4\sin^{2}(\pi f\Delta t)}}\\ & =-\sin(\pi f\Delta t) \end{array}$$

Let πfΔt=α, then according to Equations (11) and (12), sinθ=cos(α), cosθ=−sin(α). Considering 0<fΔt=Δt/T<1, there is θ=π/2+α according to the theory of trigonometric function. Then the phase of jitter displacement can be given as

(13) $$\varphi_{d}=\varphi_{r}-\theta =\varphi_{r}-\pi /2-\pi f\Delta t$$

Therefore, the quantitative relationship between the jitter displacement and relative registration error obtained from parallax images is determined and the jitter displacement estimation model is also established. After the relative registration error is obtained, components of jitter displacement including amplitude $A_{d}$ and phase φ can be estimated by Equations (10) and (13) respectively, and the frequency of jitter displacement is equal to the frequency of relative registration error. By substituting Equations (10) and (13) into Equation (3), the jitter displacement can be represented as

(14) $$d(t)=\frac{A_{r}}{2\sin(\pi f\Delta t)}\cdot \sin(2\pi ft+\varphi_{r}-\pi /2-\pi f\Delta t)$$

where $A_{r}$, f, $\varphi_{r}$ and Δt is the amplitude, frequency, phase and imaging time interval of the relative registration error between parallax images.

If SJ contains multiple frequencies, each component of the jitter displacement can be estimated from the corresponding component of the relative registration error [19].

### 2.2. Workflow of Jitter Displacement Estimation and Validation

**Figure 1:**
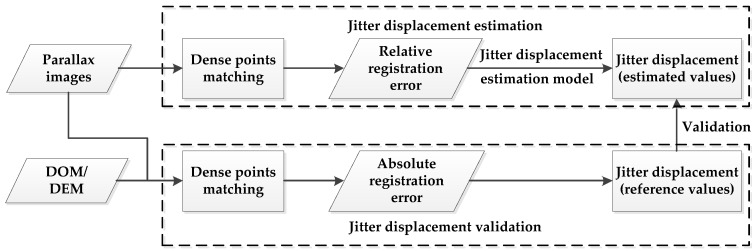
Flow chart of jitter estimation and validation.

The jitter displacement estimation and validation are carried out based on dense matching between parallax images. Both parallax images and corresponding high accuracy ground reference data (Digital Orthophoto Map (DOM) or Digital Elevation Model (DEM)) are used to do the jitter displacement estimation and validation. The workflow is shown in Figure 1.

(1) Jitter displacement estimation

Jitter displacement estimation is carried out based on the relative registration error between parallax images, where the relative registration error is computed by dense points matching. In matching, the correlation matching is used to get the initial values of corresponding points and the least squared matching is further used to obtain more precise positions of corresponding points. Then certain thresholds of coordinate differences for corresponding points are selected to filter out the mismatching points. In this paper, three times of root mean square error are chosen. After filtering mismatching points, coordinate differences of remaining corresponding points are fitted using sinusoidal function by the least squared method as the relative registration error curve. Finally, the estimated values of jitter displacement are estimated using the parameters of the fitted relative registration error curve including amplitude, frequency and phase, according to Equation (14).

(2) Jitter displacement validation

Jitter displacement validation is carried out based on the registration error between parallax image (panchromatic image or some band of multispectral image) and reference DOM data, i.e., absolute registration error. Because of the high accuracy of the reference DOM data, it can be considered as a true reflection of the ground scene, i.e., ground truth. The registration error between parallax image and reference DOM data is thus the absolute registration error. It is also an exact measurement of the jitter displacement since there should be no registration error between parallax image and reference DOM data if there is no satellite jitter. Therefore, the absolute registration error can reflect the jitter displacement in some imaging time interval of the satellite and is used as a validation of jitter displacement in this paper.

The absolute registration error between parallax image and reference DOM data is also computed by dense points matching. Parallax image should be rectified first using rational function coefficients (RPCs) and high accuracy DEM data. Then a dense points matching is conducted between the rectified parallax image and reference DOM data. Similarly, mismatching points in obtained corresponding points are also filtered out. Coordinate differences of remaining corresponding points are also fitted using sinusoidal function by the least squared method. Parameters of the fitted curve including frequency, amplitude and phase are considered as the reference values of jitter displacement and are used to evaluate the accuracy of the estimated values of jitter displacement purely based on parallax images.

## 3. Experiments and Discussion

Both simulation experiments and real image experiments were carried out to validate the feasibility and effectiveness of utilizing the built jitter displacement estimation model to do the jitter estimation. Simulation experiments were carried out to analyze the change rule of jitter displacement at different imaging time intervals and to verify the built jitter displacement model. Real image experiments were conducted according to the workflow in Figure 1 to further evaluate the performance of the jitter displacement estimation based on the built model.

### 3.1. Simulation Experiments

Simulation experiments were used to verify whether the relationship between jitter displacement curves and registration error curves satisfied the built model given by Equation (14) if there was a jitter displacement with a single sin function. Simulation experiments were carried out with different Δt (Δt=0.1T,0.2T...0.9T) and time *t* was a range from 0 to 2.5 s. Assuming $A=1^{\prime \prime }$, f=1 Hz, i.e., T=1 s, $\varphi_{d}=0\;rad$, focal length $f_{c}$ is 2 m, and the pixel size p is $2\times 10^{-5}$ m, then according to Equation (3), d(t) is determined and $A_{d}$ is 0.4848 pixel. According to Equation (4), if Δt is determined, r(t) is also determined. Therefore, it can be verified whether the relationship between d(t) and r(t)satisfies the built model given by Equation (14) in the following three aspects:

(1) Whether the frequency of d(t) is consistent with the frequency of r(t);
(2) Whether the relationship between amplitudes of d(t) and r(t) satisfies the Equation (10);
(3) Whether the relationship between phases of d(t) and r(t) satisfies Equation (13).

**Figure 2:**
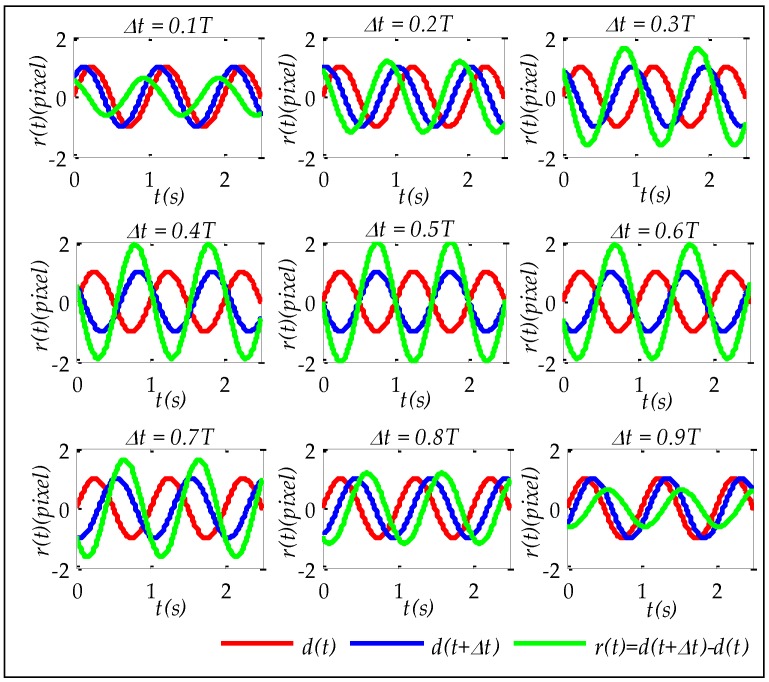
The simulated jitter displacement curves and registration error curves at different imaging time intervals.

**Figure 3:**
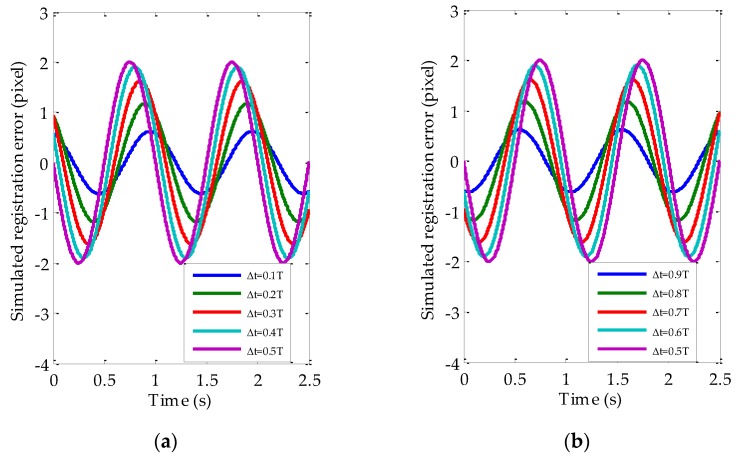
The simulated registration error curves caused by satellite jitter (SJ) at different imaging time intervals: (**a**) Δt=0.1T,0.2T...0.5T; (**b**) Δt=0.5T,0.6T...0.9T.

The simulated d(t) and r(t) with different Δt are shown in Figure 2. It is obvious that r(t) has same frequency as d(t). The amplitudes and phases of simulated r(t) are as shown in Table 1 and Table 2 respectively. The letter ‘Y’ in Table 1 and Table 2 means simulation results are satisfied with the corresponding equations, i.e., Equations (10) and (13) respectively. In addition, Figure 3 shows the simulated r(t) at different Δt. It is obvious that r(t) has a local maximum when Δt is equal to 0.5T. It is also easy to explain using Equation (10). All of these validate the feasibility of utilizing the built jitter displacement estimation model to do the jitter estimation.

### 3.2. Real Image Experiments

Jitter displacements caused by SJ are in both cross-track and along-track directions. Because jitter displacements in along-track direction are covered up by errors caused by the terrain factor, jitter displacements in cross-track direction are usually more striking than those in the along-track direction [16]. Therefore, jitter displacements in cross-track direction are used to evaluate the performance of the jitter displacement estimation based on the built model. The presented data include multispectral images of ZY-3 and images from the High Resolution (HR) camera of ZY1-02C.

#### 3.2.1. Experiment Using Multispectral Images of ZY-3 Satellite

**Figure 4:**
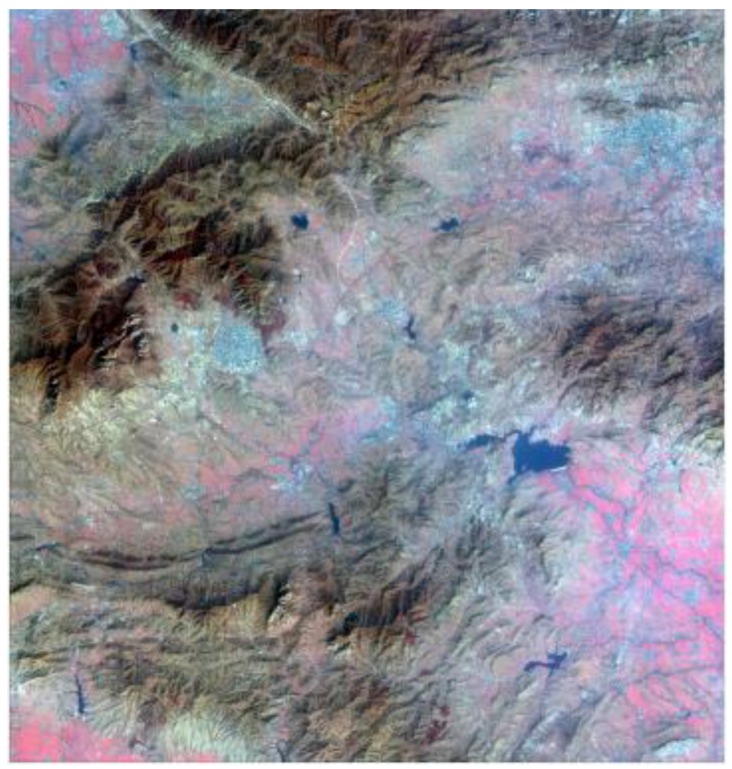
Multispectral image of ZY-3 satellite.

The presented multispectral image of ZY-3 is a level 1A product which is generated by radiometric correction and sensor correction. As shown in Figure 4, the image was captured on 3 February 2012, covering Dengfeng, Henan province, China. The image size is 8813 by 9307 pixels with a ground sampling distance (GSD) of 5.8 m. The corresponding high accuracy ground reference data include DOM with a plane accuracy of 1 m and DEM data with an elevation accuracy of 2 m.

The multispectral camera of ZY-3 satellite has four bands including blue (B1), green (B2), red (B3), and near-infrared (B4), and the corresponding CCD arrays are parallel to each other [18]. The integration time of each scanning line in a multispectral image is about 0.8 ms. The physical distance between B1 and B2 is 152 lines, and the physical distances between B2 and B3 and B3 and B4 are both 128 lines [22]. This means the imaging time interval between different adjacent bands is either 121.6 ms or 120.4 ms. Considering the difference of visible band and near-infrared band, B1, B2 and B3 were chosen to conduct the experiment. Then multispectral bands were divided into three kinds of combinations (B1-B2, B2-B3, B1-B3) to compute registration error at different imaging time intervals since the relative registration error caused by SJ varied with the imaging time interval.

**Figure 5:**
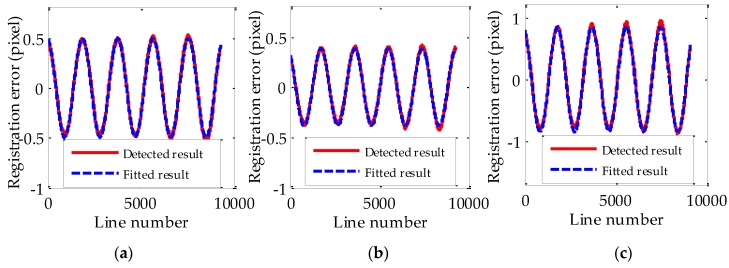
The relative registration error curves of different band combinations (**a**) B1-B2; (**b**) B2-B3; (**c**) B1-B3.

The detected and fitted relative registration error curves of different band combinations are shown in Figure 5. In Figure 5, the red and blue curves represent detected and fitted registration error curves respectively. Table 3 shows the detailed fitted parameters of relative registration error curves and the corresponding fitting residuals are shown in Table 4. The jitter displacement can be obtained based on the jitter displacement estimation model using this information. The detailed estimated parameters of jitter displacement are shown in Table 5. In Table 5, the estimated parameters of jitter displacement by different band combinations are very similar and the average amplitudes and frequencies of the estimated values of jitter displacement are around 0.9463 pixels and 0.6568 Hz, respectively.

**Figure 6:**
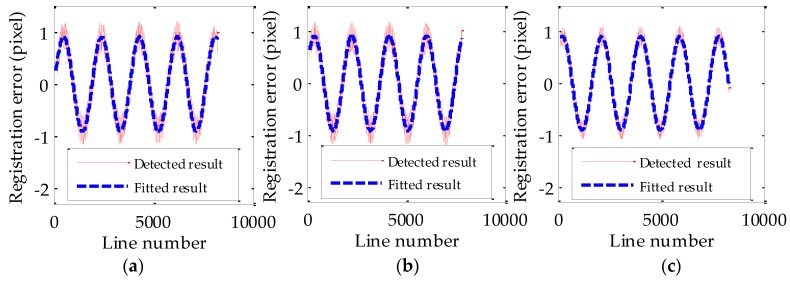
The absolute registration error curves of different bands (**a**) B1; (**b**) B2; (**c**) B3.

Reference data (DOM/DEM with high accuracy) are further utilized to obtain the absolute registration error curve as the reference values of jitter displacement in order to validate the accuracy of estimated values of jitter displacement. The detected and fitted results of absolute registration error curves obtained based on high accuracy ground reference data are shown in Figure 6. The detected result is shown as a red curve in Figure 6. The blue curve in Figure 6 is the fitted result based on the detected result and the corresponding fitting residuals of absolute registration error curves are shown in Table 6. The parameters of the fitted curve of absolute registration error (blue curve in Figure 6) are considered as the reference values of jitter displacement and shown in Table 5. In Table 5, the reference values of jitter displacement computed by different bands are also very similar and the amplitudes and frequencies of the reference values of jitter displacement are around 0.9191 pixels and 0.6561 Hz, respectively. The evaluation of the accuracy of jitter displacement estimation was also conducted, and is shown in Table 5. Obviously, the estimated values of jitter displacement are very close to the reference values. The estimated frequencies of jitter displacement by different band combinations are almost identical to the reference values. The average relative error is 0.11% and the maximum relative error is 0.23%. The estimated amplitudes of jitter displacement by different band combinations are also similar to the reference values and vary slightly. The average relative error is 2.96% and the maximum relative error is 5.37%. The band combination of B2-B3 achieves the highest estimation accuracy. Therefore, the built jitter displacement estimation model is suitable to estimate the jitter displacement for multispectral images of ZY-3 and B2-B3 is the optimal band combination for jitter displacement estimation.

#### 3.2.2. Experiment Using HR Images of ZY1-02C

**Figure 7:**
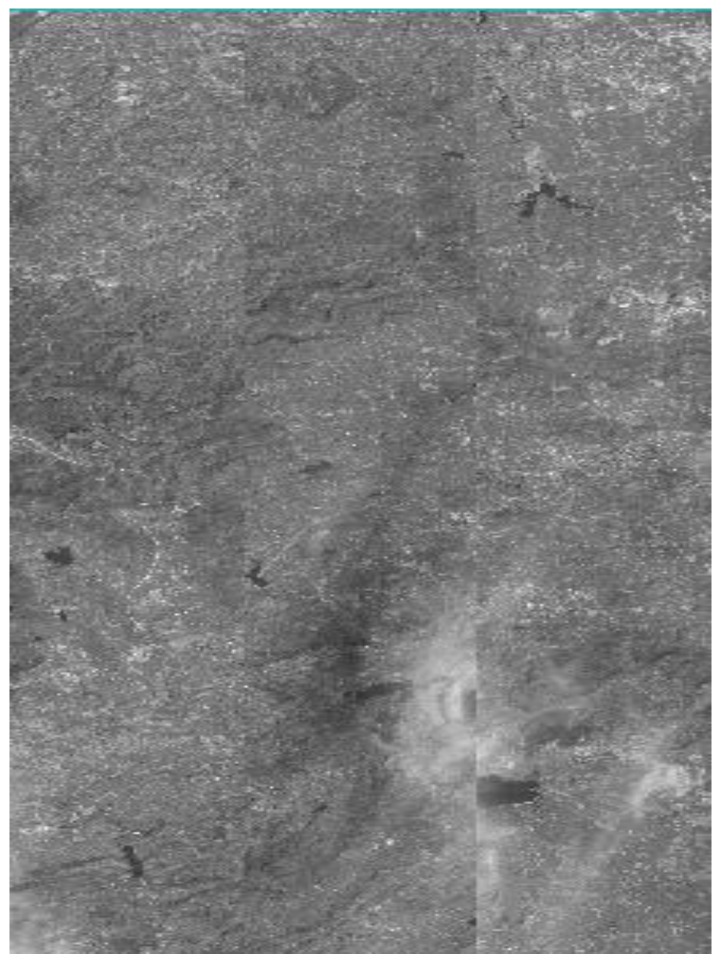
High Resolution (HR) image of ZY1-02C.

The presented HR image of ZY1-02C is a level 0 product including rational function coefficients. As shown in Figure 7, the image was captured on 13 April 2012, covering Dengfeng area, Henan province, China. The image size is 4096 by 27,575 pixels with a GSD of 2.36 m. The HR camera consists of three non-linear CCD arrays (CCD1, CCD2, CCD3), which are staggered on the focal plane along the track. The interval of along-track is about 2600 pixels and overlap of cross-track is about 30 pixels in the adjacent CCD arrays. The exposure time of each scanning line is approximately 0.35 ms, that is to say, the imaging time interval between adjacent CCD arrays is about 0.91 s.

**Figure 8:**
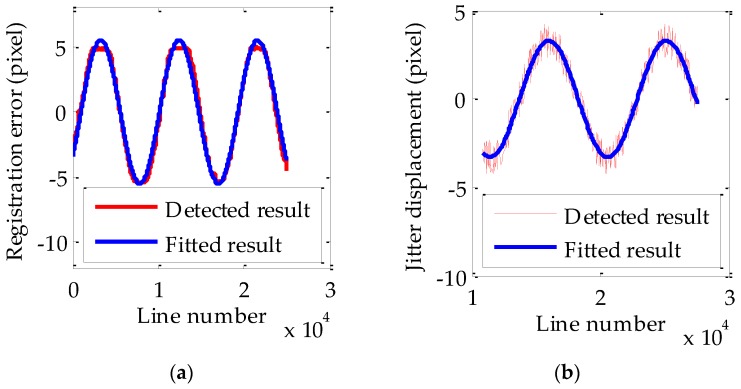
(**a**) The relative registration error curve between CCD1 and CCD2; (**b**) The absolute registration error curve of CCD1.

The process is similar to the estimation and validation using multispectral images. The detected and fitted relative registration error curve between CCD1 and CCD2 are shown in Figure 8a and the corresponding fitting residual of relative registration error curve is shown in Table 7. In Figure 8, the red and blue curves represent detected and fitted registration error curves respectively. The detailed estimated parameters of jitter displacement obtained based on the jitter displacement estimation model are given in Table 8. The amplitudes and frequencies of the estimated values of jitter displacement are around 3.5643 pixels and 0.3112 Hz, respectively. Reference data (DOM/DEM with high accuracy) are also used to get the absolute registration error curve as reference values of jitter displacement to verify the accuracy of estimated values of jitter displacement. Figure 8b shows the detected and fitted absolute registration error curve of CCD1 and the corresponding fitting residual of absolute registration error curve is also shown in Table 7. The parameters of the fitted curve are considered as the reference values of jitter displacement and also shown in Table 8. The amplitudes and frequencies of the reference values of jitter displacement are around 3.2863 pixels and 0.3123 Hz, respectively. The evaluation about the accuracy of jitter displacement estimation is also shown in Table 8. Obviously, the estimated values of jitter displacement are very close to the reference values. The relative error of the estimated amplitude is 8.46% and the relative error of the estimated frequency is 0.35%. Therefore, the built jitter displacement estimation model is also suitable to estimate the jitter displacement for HR images of ZY1-02C. Of course, 8.46% as a max error is relatively large. The relative error may be caused by matching accuracy and fitting errors. Furthermore, satellite jitter is complex and may contain multiple frequencies. Since the obtained relative registration error curves show obvious single frequency characteristics, they are processed in this work by the single frequency jitter displacement estimation model. In this procedure, error also may be introduced.

## 4. Conclusions

In this paper, the feasibility of jitter displacement estimation only using parallax images was analyzed in theory. Then the jitter displacement estimation model was built based on the quantitative relationship between the jitter displacement and the relative registration error obtained from parallax images. Finally, simulation experiments analyzed the change rule of jitter displacement estimated by registration error at different imaging time intervals and verified the feasibility of using the built jitter displacement model to estimate the jitter displacement. Real image experiments further evaluated the performance of the jitter displacement estimation based on the built model. In real image experiments, two typical images including multispectral images of ZY-3 satellite and HR images of ZY1-02C satellite were chosen as experimental data and high accuracy ground reference data were also used to evaluate the accuracy of the estimation. Experimental results indicated that the built jitter displacement estimation model can estimate the jitter displacement accurately and may be used to do the jitter detection for other sensors with parallax images.

This paper summarizes the work performed to improve upon our previous works [18,19], and includes three key developments:

(1) The deduced quantitative relationship between the jitter displacement and relative registration error obtained from parallax images in the presented paper is more accurate than our previous work. In our previous works, the relationship has only been approximated. However, the present work deduced an accurate quantitative relationship between the jitter displacement and relative registration error obtained from parallax images without any approximation.
(2) The deduced quantitative relationship between the jitter displacement and relative registration error obtained from parallax images in the presented paper is more robust than our previous work. The deduced quantitative relationship in our previous work is not an accurate expression, as its accuracy decreased when the imaging time interval increased. Obviously, such a relationship is not suitable to parallax images with large imaging time intervals such as those from the High Resolution camera of ZY1-02C. In the presented paper, the deduced quantitative relationship is accurate without any approximation. It is suitable to parallax images with any imaging time interval. Two typical images, the multispectral images of ZY-3 satellite (with a short imaging time interval between different adjacent bands of either 121.6 ms or 120.4 ms) and the HR images of ZY1-02C satellite (with a long imaging time interval between adjacent CCD arrays of about 0.91 s), were chosen as experimental data and validated the deduced relationship.
(3) High accuracy ground reference data were used to evaluate the accuracy of the jitter displacement estimation in real image experiments in the presented paper. It is an objective method. In our previous work, the accuracy of the jitter displacement estimation in real image experiments wasn’t evaluated. Other references also didn’t evaluate the accuracy of the jitter displacement detection. Generally, they evaluated the images after jitter correction [3].

Future work must also determine whether the integration stage has a certain impact on the accuracy of the jitter displacement estimation. Whether the proposed jitter estimation approach is appropriate to estimate these high frequency satellite jitters also needs to be confirmed experimentally. More accurate fitting methods may also improve the performance of the jitter displacement estimation. These will be addressed in our future work.

## Figures and Tables

**Table 1:** The amplitudes of simulated jitter displacements and registration errors at different imaging time intervals.

Simulation	0.1T	0.2T	0.3T	0.4T	0.5T	0.6T	0.7T	0.8T	0.9T
$A_{d}$	0.4848	0.4848	0.4848	0.4848	0.4848	0.4848	0.4848	0.4848	0.4848
$A_{r}$	0.29960	0.56996	0.7844	0.92211	0.96960	0.9221	0.78440	0.56996	0.2996
$A_{r}=2A_{d}\sin(\pi f\Delta t)$	Y	Y	Y	Y	Y	Y	Y	Y	Y

**Table 2:** The phases of simulated jitter displacements and registration errors at different imaging time intervals.

Simulation	0.1T	0.2T	0.3T	0.4T	0.5T	0.6T	0.7T	0.8T	0.9T
$\varphi_{d}$	0	0	0	0	0	0	0	0	0
$\varphi_{r}$	1.8850	2.1991	2.5133	2.8274	3.1416	3.4558	3.7699	4.0841	4.3982
$\varphi_{d}=\varphi_{r}-\pi /2-\pi f\Delta t$	Y	Y	Y	Y	Y	Y	Y	Y	Y

**Table 3:** The parameters of registration error curves of different band combinations.

Band Combination	Amplitude/Pixel	Frequency/Hz	Phase/Rad	Time Interval/ms
B1-B2	0.4941	0.6567	1.6854	121.6
B2-B3	0.3830	0.6566	2.1558	102.4
B1-B3	0.8045	0.6570	1.8928	224.0

**Table 4:** Fitting residuals of relative registration error curves of different band combinations.

Band	Max/Pixel	RMSE
B1-B2	0.0758	4.1023 × 10^−4^
B2-B3	0.0557	2.0165 × 10^−4^
B1-B3	0.1003	0.0011

**Table 5:** The comparison of parameters of jitter displacement for multispectral images of ZY-3.

	Estimated Values			Reference Values			
Band Combination	Amplitude /Pixel	Frequency /Hz	Band	Amplitude /Pixel	Frequency /Hz	Relative Error (Amplitude)	Relative Error (Frequency)
B1-B2	0.9911	0.6567	B1	0.9406	0.6552	5.37%	0.23%
B2-B3	0.9096	0.6566	B2	0.9145	0.6573	0.54%	0.11%
B1-B3	0.9383	0.6570	B3	0.9022	0.6558	4.00%	0.18%
average	0.9463	0.6568	average	0.9191	0.6561	2.96%	0.11%

**Table 6:** Fitting residuals of absolute registration error curves of different bands.

Band	Max/Pixel	RMSE
B1	0.3265	0.0234
B2	0.3251	0.0248
B3	0.2232	0.0122

**Table 7:** Fitting residuals of registration error curves.

Band	Max/Pixel	RMSE
CCD1-CCD2	1.1391	0.1827
CCD1	1.0861	0.3128

**Table 8:** The comparison of parameters of jitter displacement for HR images of ZY1-02C.

	Estimated Values			Reference Values			
CCD Combination	Amplitude /Pixel	Frequency /Hz	CCD	Amplitude /Pixel	Frequency /Hz	Relative Error (Amplitude)	Relative Error (Frequency)
CCD1-CCD2	3.5643	0.3112	CCD1	3.2863	0.3123	8.46%	0.35%
